# An Open-Source ABAQUS Plug-In for Delamination Analysis of 3D Printed Composites

**DOI:** 10.3390/polym15092171

**Published:** 2023-05-02

**Authors:** Efstratios Polyzos, Danny Van Hemelrijck, Lincy Pyl

**Affiliations:** Department of Mechanics of Materials and Constructions, Vrije Universiteit Brussel (VUB), Pleinlaan 2, BE-1050 Brussels, Belgium; danny.van.hemelrijck@vub.be (D.V.H.);

**Keywords:** finite element modeling, stochastic modeling, (3D printed) composite laminates, delamination, ABAQUS plug-in

## Abstract

This article presents the development and implementation of the Delamination Plug-in, an open-source tool for modeling delamination tests in the ABAQUS software. Specifically designed for stochastic modeling of 3D printed composites, the plug-in combines the benefits of the graphical user interface (GUI) and the programming of commercial finite element (FE) software. The Delamination Plug-in offers an effortless alternative to the time-consuming analytical modeling and GUI work involved in delamination tests and includes algorithms for several tests, such as the double cantilever beam, end-loaded split, end-notched flexure, and modified end-loaded split tests, solved using the virtual crack closure technique and the cohesive zone method. It enables the user to develop simulations for both simple symmetric laminates and generally layered laminates with additional thermal stresses. The applicability of the tool is demonstrated through its use in two distinct delamination problems, one for conventional and one for 3D printed composite laminates, and its results are compared to analytical models and experimental data from the open literature. The results demonstrate that the Delamination Plug-in is efficient and applicable for such materials. This establishes the tool as an important means of automating delamination analysis and for the development and testing of 3D printed composites, making it a valuable tool for both researchers and industry professionals.

## 1. Introduction

Composite laminates are widely used in various industries [1], including aerospace [2], civil engineering [3], marine engineering [4], and automotive [5]. They offer many benefits, such as increased specific properties, but are susceptible to insidious failure modes, particularly delamination, which is one of the most common failure modes for composite laminates [6]. Therefore, studying delamination is crucial for their safe application.

Numerous studies have been conducted on delamination mechanisms, and various standard organizations, such as ASTM and ISO, have developed representative test methods for characterizing the fracture toughness of unidirectional polymeric composites [7,8,9,10,11,12]. In these tests, the fracture toughness is typically equated to the Strain energy release rate (SERR).

Common delamination tests include the double cantilever beam (DCB), the end-loaded split (ELS), the end-notched flexure (ENF), and the modified end-loaded split (MELS) test. The DCB test is a common delamination test used for measuring mode I (opening mode) SERR [13]. The ELS [14] and the ENF [15] tests are used for measuring mode II (in-plane shear) SERR. Although not standardized, the MELS [16] test has been explored for measuring mixed-mode delamination.

Delamination tests are commonly conducted on symmetric laminates through standardized processes. Nevertheless, they can also be performed to measure the SERR of generally layered (i.e., non-symmetric and unbalanced) laminates [5,17]. However, especially in the latter case, the SERR is influenced by various factors, such as the applied load, the temperature difference between operation and manufacturing, the moisture conditions, and the geometric complexity of the structure [4]. The geometric complexity, in particular, can cause extension-bending coupling phenomena and residual thermal stresses, significantly affecting the prediction of the SERR.

Using simple theories in the standards is inadequate to address such complex cases, and specific analytical handling utilizing innovative bi-layer interface theories is required to accurately capture the SERR [18,19]. These theories describe the displacements and rotations at the crack tip, which are utilized to evaluate the SERR. However, direct implementation of the analytical formulas from these theories can be challenging, as differential equations up to sixth order appear [18,20].

A more effortless solution to modeling delamination problems can be achieved by using commercial finite element (FE) software, such as the widely used ABAQUS software [21]. The software includes a graphical user interface (GUI) for geometry generation, partitioning and meshing and solving structural/thermal problems. When multiple layers and materials are involved, simulating via the GUI becomes much more time-consuming, since the geometry has to be partitioned separately for each layer and material. Furthermore, the process becomes complicated when stochastic models that require the successive solution of dozens or hundreds of models are considered, making it impossible to use the existing GUI.

Stochastic models are important in the study of the delamination of 3D printed composite laminates [22,23]. The 3D printed composite laminates are produced by the sequential printing of laminas and can offer design freedom and increased stiffness and toughness. However, due to the printing process, the bonding between the laminas in the stacking sequence can be inconsistent, thus leading to high scattering in the SERR of such structures. Additionally, dimensional uncertainties that appear due to the manufacturing process can also cause scattering of the SERR. These uncertainties can be described by stochastic models, the development of which, as explained above, cannot be accomplished via the existing GUI.

In software such as ABAQUS, the modeling of such structures can be achieved via scripting in object-oriented Python, which allows for full parametrization and optimization of the software’s capabilities. However, this task is non-trivial and requires programming experience with concurrent knowledge of the background FE algorithm. Furthermore, scripting lacks the ease of use of the GUI.

Plug-ins combine the efficiency of scripting and the practicality of the GUI by developing a tailored algorithm for the solution of distinct problems, with the aim of automatizing the process. The implementation of this algorithm is achieved via a custom GUI panel (the plug-in) that aids the user’s interaction with the background script. The user can then provide parameters to the script via the plug-in and create, solve, and process results much faster and without investing in studying or developing the background script.

Various plug-ins have been developed for solving problems in micromechanics [24,25,26], viscoelasticity [27], damage [28], and fatigue [29] analyses. However, to the best of the authors’ knowledge, no such tools exist for the simulation of delamination tests.

Considering the above, this paper aims to develop the **Delamination Plug-in**, an accurate and practical means of modeling delamination tests in the ABAQUS software. The innovative tool combines the advantages of the GUI and the programming of commercial FE software and offers an effortless alternative to the time-consuming analytical modeling and GUI work needed when performing delamination tests. The plug-in allows for simulations of delamination tests for generally layered laminates with additional thermal stresses and, furthermore, drastically simplifies the stochastic modeling of 3D printed laminates. The background algorithms of the open-source tool are provided for specialized individual applications and modifications. Thus, the Delamination Plug-in can be considered as a foundation stone of the automatization of delamination analysis and can accompany the analytical and experimental work of researchers and industry professionals in the domain.

This work is structured as follows. Section 2 introduces the important background on delamination approaches that are used in numerical methods and analytical theories. Section 3 presents the process of modeling a structure using the Delamination Plug-in. Finally, Section 4 demonstrates the applicability of the Delamination Plug-in considering two case studies and offers a comparison with analytical models and experimental data.

## 2. Background on Delamination

### 2.1. Numerical Simulation

Several studies have utilized the FE method to investigate delamination problems in both one and two dimensions [30]. This research paper specifically focuses on 1D delamination. In the existing literature, two commonly used methods for defining cracks are the cohesive zone method (CZM) [31] and the virtual crack closure technique (VCCT) [32,33]. To assist with the implementation of these methods in ABAQUS and the development of a plug-in, a brief overview is provided.

The CZM was first introduced by Dugdale [34] and Barenblatt [35]. This method employs interfacial cohesive elements, positioned in the direction of the crack, to define a constitutive law for the material behavior in the crack direction. The cohesive elements combine a fracture mechanics and a stress-based formulation. To estimate the SERR, including the mode I ($G_{I}$) and mode II ($G_{II}$) components, the J-integral is used. The total SERR (*G*), which is equal to the J-integral, is given as a function of the stress (σ) and the displacement (δ) after some analytical treatment as

(1) $$G=G_{I}+G_{II}=\int_{0}^{\delta }\sigma \left(\delta \right)d\delta$$

**Figure 1:**
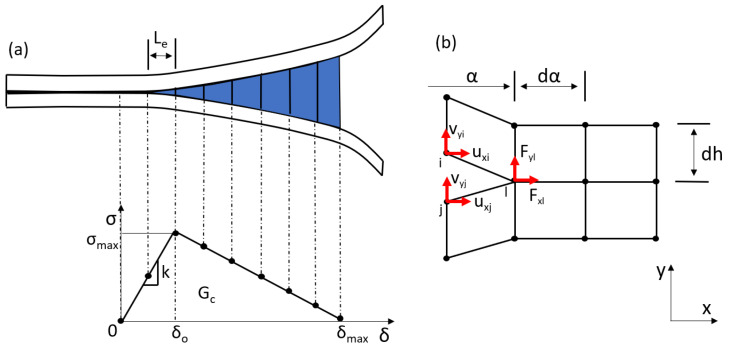
Implementation of the CZM using elements with linear softening (**a**) and the VCCT (**b**) for interfacial separation.

It is important to note that the SERR components are obtained through the integration of the corresponding stresses. In practice, the CZM is typically implemented by introducing elements with linear elastic softening behavior. The interfacial constitutive equation is illustrated in Figure 1a. To prevent relative movement of the upper and lower sub-laminates in the initial stages of the linear elastic region, a high initial penalty stiffness (*k*) is used. The linear softening part is then introduced when the stress reaches a maximum value ($\sigma_{max}$) at a displacement $\delta_{o}$, and the element contribution decreases until the area under the stress-displacement curve is equal to the critical SERR ($G_{c}$). These elements are available in the ABAQUS element library and are used in the present plug-in. To capture the proper energy dissipation, the constitutive parameters, including the size of the cohesive element ($L_{e}$) and the interface strength ($\sigma_{max}$), must be appropriately defined. For more information on the evaluation of these parameters, refer to the work by Turon et al. [36].

The VCCT, originally proposed by Rybicki and Kanninen [37], calculates the SERR *G*, and its components $G_{I}$ and $G_{II}$, using the nodal forces (*F*) and displacements (u,v) of the nodes situated near the crack tip (i,j,l). For four-node elements, the expressions for *G* and its components can be written as

(2) $$G=G_{I}+G_{II}=\frac{F_{yl}\left(v_{yj}-v_{yi}\right)}{2bd\alpha }+\frac{F_{xl}\left(u_{xj}-u_{xi}\right)}{2bd\alpha }$$

To effectively model crack propagation using the VCCT, it is necessary to use adequately refined meshes of equally sized elements in the crack direction. The mesh size should be in the range of 0.1 to 1.0 times the crack width (out-of-plane dimension). However, this requirement can lead to computationally intensive models with dense meshes. For the VCCT, the user defines the connected regions where the crack will propagate, and the nodes between the two surfaces are rigidly attached and released when the computed SERR meets a failure criterion. The B–K criterion [21,38] is typically used for this purpose. More information on the VCCT is provided in [32,33]. Figure 1b illustrates the VCCT for four-node elements.

### 2.2. Analytical Theories

Numerous analytical theories have been created to study delamination tests [19], which generally rely on 2D elasticity. The composite laminate undergoing delamination consists of two sub-laminates that are bonded on their common surface, and the crack progresses in one direction between these sub-laminates (known as 1D delamination).

Advanced delamination theories, such as the conventional composite beam theory, the shear deformable bi-layer beam theory, and the interface deformable bi-layer beam theory, assume that the sub-laminates are Timoshenko beams and are modeled using first-order shear deformation theory (FSDT). These bi-layer beam theories are described as the rigid, semi-rigid, and flexible joint deformation models, respectively. Unlike the standards-based theories [13,14,15], the advanced delamination theories are corrected for shear and can account for generally layered laminates.

The three delamination theories assume different boundary conditions between the two sub-laminates. The conventional composite beam theory considers that both the relative displacement and rotation are restricted on the interface between the two sub-laminates. The shear deformable beam theory releases the rotational degree of freedom and considers that only the relative displacement is restricted. This improves the accuracy of the theory and mitigates significant errors caused by the zero slope restrictions at the beam root [39]. Finally, the interface deformable beam theory permits both relative displacement and rotation.

The bi-layer theories describe the displacement, rotation, and stresses (shear and normal) at the bonded section. The displacement and rotation of the bonded section are then utilized in a J-integral [7] equation to compute the SERR [18,19,40].

## 3. Software Methodology

**Figure 2:**
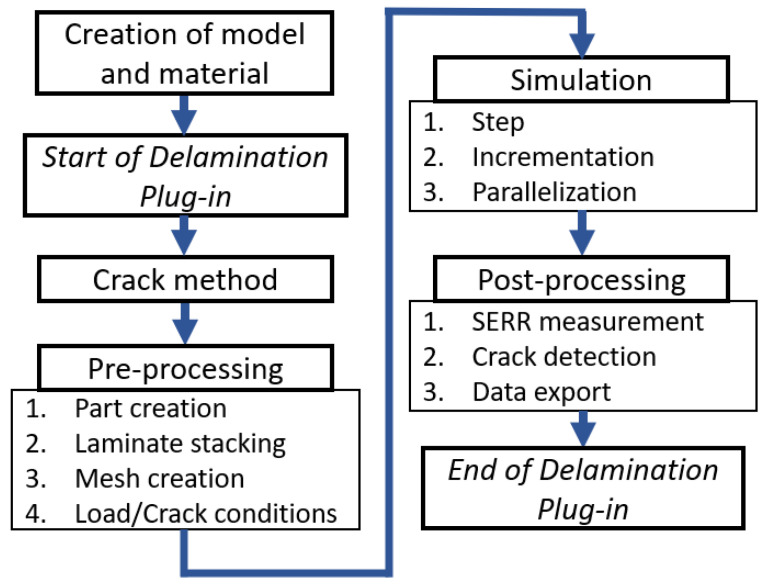
Flowchart of the software methodology.

The Delamination Plug-in offers a practical and effective 2D simulation of delamination tests. The long GUI process, from geometry preparation until the post-processing, is automated, requiring only the necessary parameters for a correct delamination simulation. The parameters will be introduced stepwise in the following sections. The software is completely written in Python 2.7 utilizing the built-in ABAQUS Python application programming interface (API). The simulation is separated into three main phases: the pre-processing, simulation, and post-processing. The parameters of the main phases are inserted using five tabs in a straightforward process. Graphical representations of the required input parameters are illustrated to aid the understanding of the user. A flowchart describing the main phases of the software is presented in Figure 2.

The Delamination Plug-in requires an active model and materials (one or more) with elastic constants defined as ‘engineering constants’ and ‘orthotropic’ thermal expansion coefficients.

**Figure 3:**
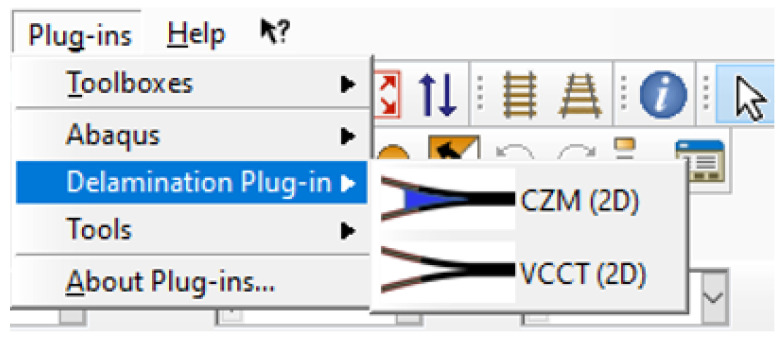
Initial dialog of the Delamination Plug-in.

The plug-in is initialized simply by placing it in the directory ‘abaqus_plugins’. The user can choose to initiate the plug-in either with CZM or VCCT (Figure 3). The two methods, the VCCT and the CZM, have been included as separate analyses due to their differences in part generation and input parameters. Different graphical illustrations have been added for both methods to ensure the correct representation of input parameters and guidance for the user. Here, the CZM is presented as the method requiring the most input parameters and the characteristics of the software are extensively described. Important differences between the CZM and the VCCT are also indicated to aid the user.

The background of the three phases is elaborated in the following section, addressing the most important modeling points.

### 3.1. Pre-Processing

**Figure 4:**
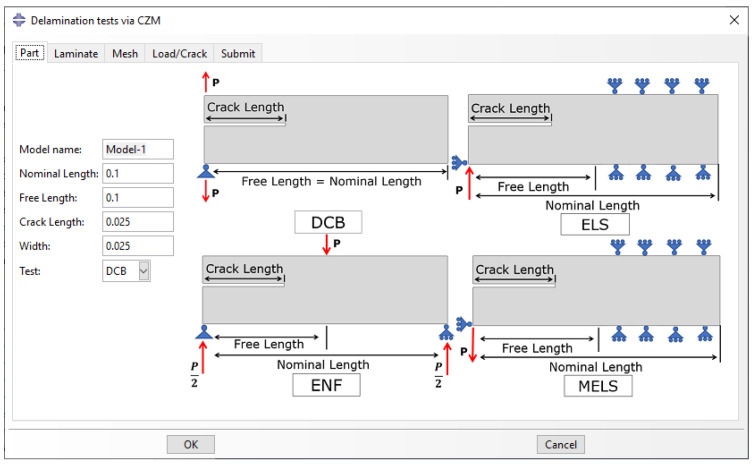
‘Part’ tab: Selection of delamination test via CZM.

In the pre-processing phase, the user creates and meshes one of the four parts available in the library: the DCB, the ELS, the ENF and the MELS. The ‘Part’ tab (Figure 4) allows the selection of one delamination test with user-defined dimensions in an active model. A 2D part as planar shell is created with the given nominal length, free length, crack length, and width (out-of-plane dimension).

**Figure 5:**
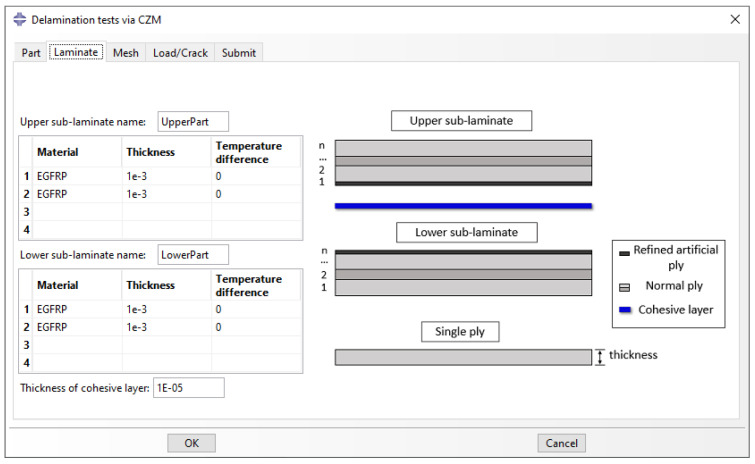
‘Laminate’ tab: Definition of laminate stacking sequence.

The laminate stacking sequence is defined in the ‘Laminate’ tab (Figure 5). The user can define the stacking sequence for both the lower and the upper sub-laminates using *n* number of plies. Different materials (column material), thicknesses (column thickness), and temperature differences (column temperature) can be given for each ply. A thin cohesive layer is defined for the CZM. Furthermore, a thin ply (refined artificial ply) is created on both sides of the user-defined crack. The refined artificial ply addresses the issue of convergence of the components of SERR for VCCT as described in Section 2. The thickness of the artificial ply needs to be such that the overall bending stiffness of the two sub-laminates remains unaffected [5]. For CZM, the refined artificial ply is used to offer progressive mesh refinement between the lower and upper sub-laminates and the cohesive layer. The addition of the artificial ply is suggested by fractographic evidence in experimental studies of delamination [32,33]. The software uses the input data to partition the geometry into separate layers and automatically assigns the material properties and temperature difference values to the partitions using ‘homogeneous sections’ in ABAQUS.

**Figure 6:**
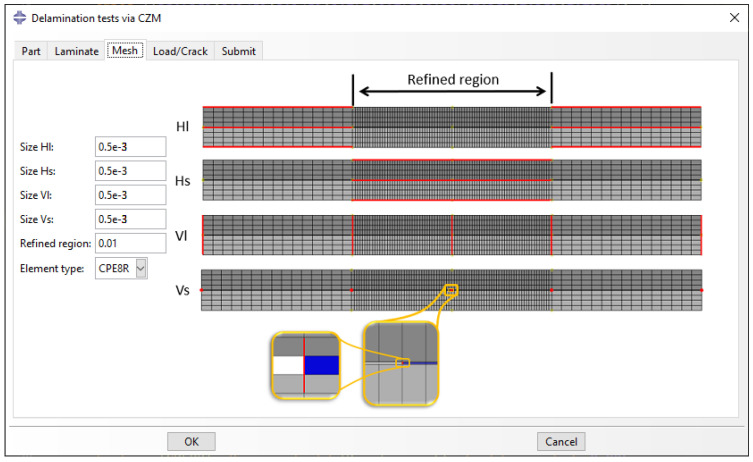
‘Mesh’ tab: Automatic meshing of the part.

The mesh parameters are defined in the ‘Mesh’ tab. In ‘Refined region’, the user specifies the length of the refined region, which is symmetric around the crack tip. The refined region is used to densify the mesh close to the crack tip, offering high accuracy and lower computational cost [5]. Four mesh size parameters are used to define the mesh of the lower and upper sub-laminates. The horizontal short ($H_{s}$) and long ($H_{l}$) edges refer to the size of the elements of the horizontal edges inside and outside the refined region, respectively. The vertical short ($V_{s}$) and long ($V_{l}$) edges refer to the size of the elements of the edges of the artificial refined ply and the normal plies. For CZM, the $V_{s}$ is additionally used for the thickness of the cohesive layer. All edges are depicted using red in Figure 6. Finally, the user can choose either plane-strain or plane-stress elements. The use of quadratic reduced-integration elements is suggested to avoid shear locking and offer faster solutions. The elements are illustrated using the terminology of ABAQUS. For instance, the plane-strain quadrilateral eight-node reduced-integration element is referred to as CPE8R. The cohesive layer for CZM is assigned by default a four-node 2D cohesive element (COH2D4), since this is the only 2D cohesive elements in the ABAQUS library.

**Figure 7:**
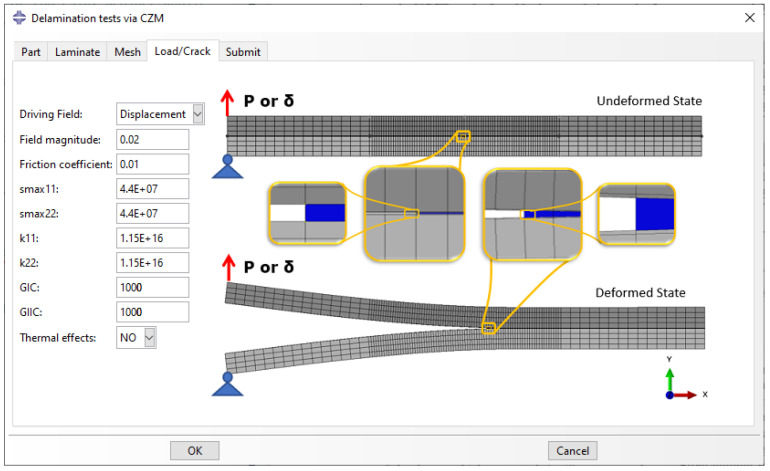
‘Load/Crack’ tab: Creation of loading conditions and interactions.

The ‘Load/Crack’ tab, illustrated in Figure 7, is used to assign the boundary conditions and the interactions between the lower and upper sub-laminates. A constant force (*P*) or displacement (δ) can be added as a driving field of specified magnitude. The contact between the two sub-laminates is modeled as ‘hard contact’ with a user-defined friction coefficient, since the friction between the two sub-laminates can affect the accuracy of SERR estimation [41]. The bonded part of the sub-laminates (and the cohesive part for the CZM) is given ‘surface-to-surface’ constraints to ensure perfect bonding between elements with uneven sizes or node number. For an almost frictionless behavior, the friction coefficient can be given a very low value. Furthermore, parameters associated with the crack initiation and propagation can be set. For the VCCT, the only necessary parameters are the mode I and II ($G_{IC}$ and $G_{IIC}$) critical SERR values. For the CZM, the parameters additionally include the stiffness penalty (*k*) and the maximum interfacial strength ($\sigma_{max}$) in the axial (11 or X) and normal (22 or Y) directions. Finally, the user can choose to activate the thermal effects and perform a thermal analysis before the mechanical one.

Even though ABAQUS offers the possibility of performing both the thermal and the mechanical analysis in the same step, here the two analyses are separated. If both analyses are performed in the same step, the initial conditions are zero, and the thermal and mechanical conditions are gradually imposed, which does not accurately represent the actual test. Therefore, performing the thermal analysis before the mechanical ensures the accurate simulation of the delamination test by using the deformation due to thermal effects as the starting point of the mechanical analysis.

### 3.2. Simulation

**Figure 8:**
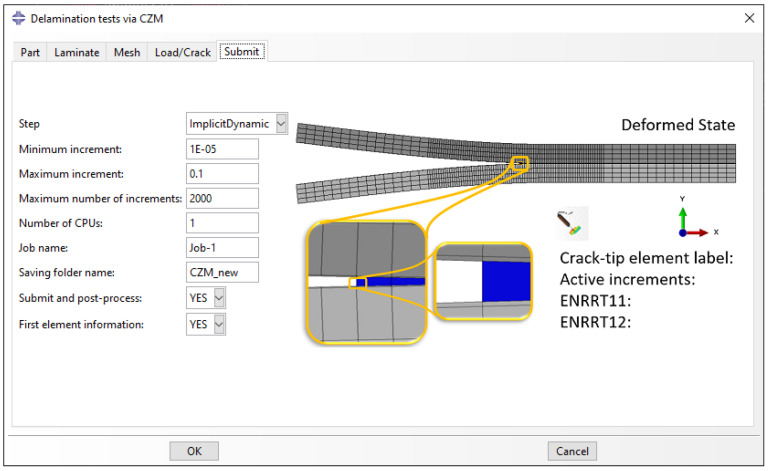
‘Submit’ tab: Simulation and post-processing parameters.

The simulation phase of the plug-in (‘Submit’ tab illustrated in Figure 8) requires the information for the step, the incrementation, and the parallelization. A static or implicit dynamic (quasi-static) step can be chosen for the mechanical analysis. The incrementation of the solver also plays a crucial role in the convergence and the solution of the delamination analysis. Therefore, the user is given the option to alter the minimum and maximum increments as well as the maximum number of increments that will be performed before the solver automatically terminates the process. Moreover, it is common that a large number of increments is used for the convergence of the solution, and including multiple CPUs is useful to reduce the computational time. Thus, the user has the possibility to choose the number of CPUs that will be adopted for the analysis. The job is automatically created under the defined job name.

The final step is to define a folder name where the post-processing output will be saved.

### 3.3. Post-Processing

The post-processing phase is initialized by activating the ‘Submit and post-process’ in the ‘Submit’ tab. Here, the process of SERR measurement and crack detection is automated and information is exported in separate CSV files containing all the time increments.

For the VCCT, the information for the nodes that belong to the bonded section of the two sub-laminates includes the debond status, the stress, the strain, and the energy release rate (ERRT from ABAQUS). Furthermore, a file containing all the input parameters used for the analysis, as well as the load, displacement, and energy values, is exported. Finally, a file containing the location of the crack tip in every increment is exported, in addition to the coordinates of each of the bonded nodes using a custom crack detection algorithm. The crack detection algorithm, compares the bonding status of the nodes and compares the time of opening. Then, it assigns the coordinates of the opening nodes to the crack tip.

For the CZM, similar information is extracted concerning the cohesive elements. The debond status, the stress, and the strain of each integration point are given for each element. Here, the ERRT is estimated using Equation (1). The information concerning the load, the displacement, the energy, and the crack is similarly exported. For the crack detection, the data of the cohesive element status are used.

Finally, information of the first node (for the VCCT) or the first element (for the CZM) in the crack direction can be conveniently exported and printed in the ‘Message Area’. The information includes the label of the first node or element, the SERR components, and the active increments, and can be used to identify whether the crack propagates immediately from the GUI. The CSV files can be used to perform separate post-process analyses and visualize the information in separate software. The output files contain the most crucial information required for delamination tests.

It is important to mention that during the use of the Delamination Plug-in, critical mistakes for the geometry creation are reported, aiding the user.

## 4. Application

Two case studies are given here to demonstrate the abilities of the Delamination Plug-in. Firstly, considering generally layered laminates with residual thermal stresses, a case study is conducted for a fiber metal laminate (FML) containing layers of aluminum and e-glass fiber reinforced polymer (EGFRP). This case study is of particular interest since it has been used before as a comparison of novel analytical methods [5,17] and it is an excellent example of a case where the geometry is complex, consisting of multiple materials and layers. Due to the complex geometry, the thermal effects lead to high residual stresses which drastically influence the predictions of the SERR.

Secondly, an example is taken from the field of 3D printed composite laminates. The performance of 3D printed laminates is highly influenced by uncertainties related to the manufacturing process. In particular, large scattering can be observed in their maximum load capacity when tested in mode I or mode II delamination, which is an indication of an inconsistent bond that forms between the laminates while printing. Furthermore, the tolerances of the 3D printing lead to uncertainties in the dimensions of the final part, which can also influence the outcome of delamination tests. The above uncertainties are characterized as dimensional and performance-related uncertainties and have been addressed for 3D printed nylon reinforced with carbon fibers in [22].

The Delamination Plug-in can be used to capture the aforementioned uncertainties and facilitate an effective stochastic simulation that would otherwise require time-consuming, repetitive work. The example used here is taken from [22], where the authors implemented the pseudo spectral projection method (PSPM), one of the two non-intrusive polynomial chaos expansion (PCE) methods, to model the load-displacement behavior of the 3D printed laminate under DCB and ELS testing. For this example, the development of the stochastic model using the Delamination Plug-in is of primary interest and it is presented and elaborated here.

### 4.1. Fiber–Metal Laminate

**Figure 9:**
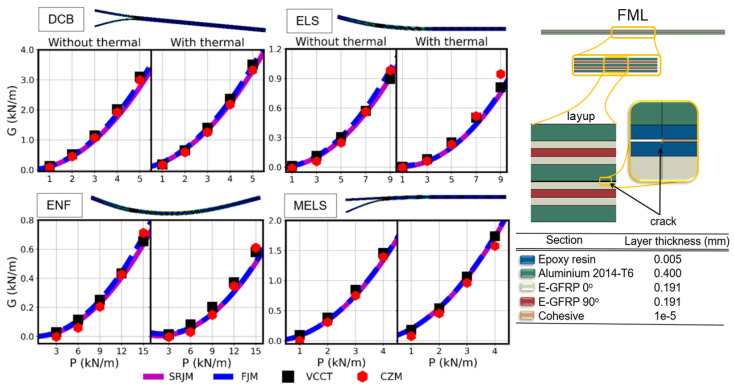
SERR versus load curve of delamination tests of FML with and without thermal effects and stacking sequence and geometry of the FML.

The FML, illustrated in Figure 9, comprises several thin layers of aluminum 2014-T6 alternately bonded with EGFRP. In this study, it is subjected to DCB, ENF, ELS, and MELS tests using both the VCCT and the CZM. The material information of the study of Bhat and Narayanan [42] for the EGFRP and the GLARE is utilized and summarized in Table 1. All simulations are performed using a crack length of 25 mm, and a nominal length of 100 mm. The free length is chosen for the DCB as 100 mm and for the ELS, ENF and MELS as 50 mm.

Mesh convergence studies are conducted to define the appropriate mesh used for the simulations with and without thermal effects. As an example, the mesh parameters associated with the DCB test without thermal effects are specified in mm as 2.5 × 10^−4^, 2.5 × 10^−5^, 2.5 × 10^−4^, 5 × 10^−6^ for the Hl, Hs, Vl, and Vs, respectively. All simulations are performed using a ‘Force’ driving field and the critical values of SERR ($G_{IC}$ and $G_{IIC}$) are given a high value (10,000 N/m) to prevent the crack from propagating so that the SERR is measured for a stationary crack. The parameters associated with the use of the CZM are defined using the methodology of Turon et al. [36]. The simulation is performed using an implicit dynamic (quasi-static) step. Finally, important to note is that the driving force ‘P’ is reduced over the specimens’ width and the temperature difference is taken as −135 degrees, similarly to the study of Tsokanas and Loutas [5].

The numerical models are validated using the analytical solutions of the semi-rigid joint model (SRJM) and the flexible joint model (FJM) of Qiao and Wang from their pioneering analytical work [18]. These models are chosen since they represent the exact analytical solution of the delamination problem.

The SRJM is the implementation of the shear deformable beam theory (see Section 2) and considers that the two bonded sub-laminates have zero relative displacements, but the relative rotation is allowed. This avoids errors in the prediction of the SERR that can be introduced due to the assumptions of zero rotations at the tip of the crack [39]. The relative displacements are allowed in the FJM, which is an implementation of the interface deformable beam theory (see Section 2). Here, a series of springs is added between the two sub-laminates to simulate the relative translations between the two sub-laminates. This implies that the FJM is more general than the SRJM, but both models have been extensively used in various applications due to their overall accuracy [5,18,19,22,43,44].

The measured data of the SERR are extracted and compared with the analytical models. The total SERR (*G*) versus the load (*P*) is illustrated in Figure 9 in separate graphs for the case with and without residual thermal stresses. The results indicate an overall great agreement between the numerical results and the analytical solutions. Furthermore, note that the results are in agreement with the solutions presented in the work of Tsokanas and Loutas [5] for the DCB and ENF tests.

### 4.2. Stochastic Modeling of 3D Printed Composites

The implementation of the PCE method is similar to the Monte Carlo (MC) method. Both methods require the generation of points and the solution of a deterministic model (here the FE model of the delaminating specimen) on each point. These points can be, for example, groups of material and geometry properties that are generated via the probability distribution of each property. The generation of points for the PSPM method can be achieved using open-source libraries such as chaospy [45] in Python, as in [22], and it is independent of the simulation of the deterministic model performed in ABAQUS. The number of points can be between a few dozens and a few hundred, with one simulation required for every point.

Considering the above, performing a stochastic simulation necessitates first generating the points and then creating an FE model for each point. However, if performed manually, this process is time-consuming. Hence, it is automatized here using the Delamination Plug-in.

The process to develop each deterministic model is identical to the one presented in Section 4.1 in terms of the definition of the parameters, meshing, and solving the model. Therefore, only the part referring to the stochastic modeling using the Delamination Plug-in is elaborated here.

**Figure 10:**
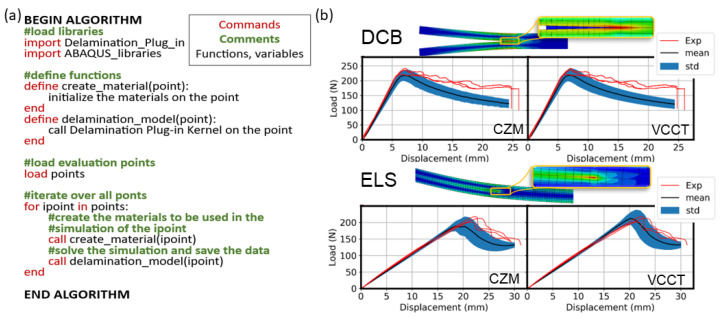
(**a**) Pseudocode used for the stochastic simulation. (**b**) Stress versus displacement curve of delamination tests of 3D printed nylon reinforced with continuous carbon fibers.

Figure 10a illustrates the pseudocode that is used for the stochastic simulation. Firstly, the Delamination Plug-in is loaded together with the libraries of ABAQUS. Then, two functions are defined, the ‘define_material’ and the ‘delamination_model’ function. The ‘define_material’ function is used to initialize the materials that will be included in the simulation using the Delamination Plug-in. The ‘delamination_model’ function calls the kernel of the Delamination Plug-in as a simple function with the *i*th point argument. Finally, an iterative ‘for’ loop is used to call the two functions and solve the delamination problem for all points. This process allows one to successively solve all stochastically generated FE models. The solutions are stored in separate folders which can be accessed later to visualize and process the results. In view of the above, using the Delamination-Plug in, the process of performing stochastic simulations can be largely simplified, since the generation and the solution of the numerous FE models reduces to calling a single function, the ‘delamination_model’ function.

The stochastic modeling is applied here for the DCB and the ELS test of symmetric 3D printed laminates of nylon reinforced with carbon fibers. The stochastic parameters (the crack length α and the maximum load $P_{max}$) that are used for the simulations are summarized in Table 2 along with the remaining elastic properties that are deterministic. For the full details of the stochastic model and the specimen manufacturing and testing, the reader is referred to [22]. The results of the stochastic models developed using the CZM and the VCCT are presented in Figure 10b and compared with experimental data from [22].

For the DCB test, the results of the VCCT and the CZM present an overall agreement with the experimental data. In particular, the initial linear part of the curve and the maximum load are accurately captured. However, the propagation part (part of the curve after the maximum load that instigates crack propagation) exhibits differences. These differences are attributed to the artificial stiffening of the laminate due to fiber-bridging, as explained in [22]. For the ELS test, a very close agreement is seen with the experimental results for the CZM and the VCCT. Here, both the linear and the propagation part are captured well.

## 5. Conclusions

Understanding the delamination process of composite laminates is an important task, since delamination is one of the most prevalent failure modes. The task can be tackled either analytically or numerically. However, complex geometries with general stacking sequences and residual thermal stresses are difficult to express analytically due to the complexity of the analytical solutions. Focusing on the numerical modeling, this article addresses the development of the Delamination Plug-in, a functional and practical tool for performing simulations of common delamination tests using the ABAQUS commercial FE software.

The plug-in combines the advantages of the GUI of ABAQUS with a powerful custom kernel which drastically accelerates the pre-processing and post-processing phases. The Delamination Plug-In contains built-in geometry creation modules, adaptive user-defined meshing, and automatic generation of loading and interaction conditions. The simulation process is conducted in a straightforward manner using the VCCT or CZM. The user is offered the capability of creating customized models of simple and complex stacking sequences and visualize the data in separate software with powerful plotting tools. Stochastic modeling is also simplified, since the generation and the solution of the numerous FE models reduces to calling a single function in an iterative loop. Finally, distributing the algorithm of the Delamination Plug-in gives the opportunity to extend the capabilities of the plug-in and allows for custom development.

In conclusion, the open-source Delamination Plug-in can accompany analytical or experimental work and aid the field of delamination analysis by providing a practical and effective alternative to complex analytical models or time- consuming GUI development in FE software.

The latest version of the Delamination Plug-in can be acquired from the corresponding author following an email request.

## Figures and Tables

**Table 1:** Elastic and thermal properties of aluminium 2014-T6, EGFRP and epoxy resin.

	Aluminium	EGFRP	Epoxy Resin
$E_{11}\left(\mathrm{GPa}\right)$	72.00	38.73	3.50
$E_{22}\left(\mathrm{GPa}\right)$	72.00	6.94	3.50
$G_{12}\left(\mathrm{GPa}\right)$	27.06	2.50	1.25
$\nu_{12}\left(\right)$	0.33	0.27	0.33
$a_{11}$ ($10^{-6}$/°C)	23.00	7.26	57.50
$a_{22}$ ($10^{-6}$/°C)	23.00	37.70	57.50

**Table 2:** Stochastic parameters (crack length α and maximum load $P_{max}$) and deterministic parameters ($E_{11}$, $E_{22}$, $G_{12}$, and $\nu_{12}$) that are used for the simulations. The mean and the standard deviation are presented only for the stochastic parameters.

	$E_{11}$ (GPa)	$E_{22}$ (GPa)	$G_{12}$ (GPa)	$\nu_{12}\left(\right)$	α (mm)	$P_{\max}$ (N)
DCB	45.0	2.3	4.0	0.44	54.4 ± 2.0	225.9 ± 20.1
ELS	45.0	2.3	4.0	0.44	53.8 ± 1.2	226.6 ± 21.1

## Data Availability

The research data produced in this study can be obtained by contacting the authors.
